# Selected Aspects of the Mental Functioning of Women After Childbirth in a Hospital During a Pandemic

**DOI:** 10.3389/fpsyt.2022.846645

**Published:** 2022-05-06

**Authors:** Agnieszka Kułak-Bejda, Maryla Malinowska-Gleń, Grzegorz Bejda, Anna Slifirczyk, Napoleon Waszkiewicz

**Affiliations:** ^1^Department of Psychiatry, Medical University of Białystok, Białystok, Poland; ^2^The PhD Studies, Medical University of Białystok, Białystok, Poland; ^3^Department of Perinatology, Medical University of Bialystok, Białystok, Poland; ^4^The School of Medical Science in Białystok, Białystok, Poland; ^5^Faculty of Health Sciences, State Higher School of Pope John Paul II, Biała Podlaska, Poland

**Keywords:** COVID-19, pandemic, childbirth, emotions, motherhood

## Abstract

**Introduction:**

The COVID-19 pandemic is stressful for pregnant women, their families, and their unborn baby.

**Aim of the Study:**

The study aimed to assess the impact of a pandemic on the mental state of women after childbirth.

**Material and Methods:**

The study included 363 women hospitalized after delivery. The study used a diagnostic survey method using the proprietary questionnaire and the Family Affluence Scale (FAS), Edinburgh Postnatal Depression Scale (EPDS), Jong Gierveld Loneliness Scale (DJGLS), The Basic Hope Inventory (BHI-12), and General Self Efficacy Scale (GSES).

**Results:**

Suspicion of postpartum depression was found in 109 women (mean: 15.28 ± 2.22)—group I, and no suspicion of it in 254 subjects (mean: 6.03 ± 2.63)—group II. Mean values of the sense of loneliness in group I (27.11 ± 6.00) were higher than in group II (21.35 ± 7.02), and the basic hope—BHI-12, in group I—lower (27.92 ± 5.14) than in group II (31.75 ± 4.97). In the Generalized Own Efficacy Scale, the group I obtained lower mean values (28.07 ± 4.86 points and 5.87 ± 1.96 points) than group II (30.97 ± 3.77 points and 6.02 ± 1 points, 38 sten).

**Conclusions:**

As much as 30% of the respondents showed a risk of postpartum depression. The most felt was the limitation of family visits during the hospital stay. In addition, the respondents were most concerned about the child's health in both groups. The feeling of loneliness in group I was higher, and basic hope and generalized self-efficacy were lower than in group II. The differences between these relationships were statistically significant.

## Introduction

The period during a pandemic makes it necessary to follow the rules of a sanitary regime even though childbirth is similar to childbirth under non-pandemic conditions. However, in the context of the possibility of contracting severe acute respiratory virus syndrome coronavirus 2 (SARS-CoV-2), pregnant women may be more worried, not only for themselves and their families but also for the unborn baby.

However, in the context of the possibility of contracting SARS-CoV-2, pregnant women may be more worried—not only for themselves their families but also for the unborn baby (1). As a result, preterm labor occurred in 41% of patients, and premature rupture of fetal membranes in 19% of patients. In addition, severe internal asphyxia of the fetus was found in many patients (43%). However, a particular bias of these results cannot be ruled out because the description includes retrospective case studies. Therefore, a particular bias in the results should be considered.

The research conducted by Breslin et al. (2) indicates that 44% of deliveries are resolved by surgery. A meta-analysis by Zagiham et al. (3) showed that 92% of coronavirus 2019 (COVID-19) patients gave birth by cesarean section.

Despite different percentages, it can be clearly stated that a patient infected with SARS-CoV-2 is associated with an increased percentage of pregnancy completion by cesarean section in the general population.

It has also been demonstrated that pregnant women suffering from COVID-19 have a three times greater risk of premature delivery or pregnancy termination by cesarean section. Tests conducted on more than 2,130 pregnant women from 18 countries suggest that pregnant women with COVID-19 were more than 50% more likely to experience complications related to pregnancy compared to pregnant women not suffering from the coronavirus (4). Each woman with the coronavirus was compared to two healthy women who gave birth concurrently in the same hospital. A total of 706 pregnant women diagnosed with COVID-19 and 1,424 pregnant women diagnosed with COVID-19 were enrolled in the study, all with substantially similar demographic characteristics (mean age 30.2 ± 6.1 years). Women diagnosed with COVID-19 were more likely to develop pre-eclampsia/eclampsia, severe infections, intensive care, maternal mortality, preterm labor, severe neonatal morbidity, and perinatal morbidity and mortality.

Newborns mothers who had positive COVID-19 test results in 13% also had positive test results. Delivery by cesarean section was associated with an increased risk of a positive neonatal test result associated with pregnancy termination by cesarean section rather than by breastfeeding. Newborns of women diagnosed with COVID-19 had a significantly higher rate of severe morbidity and a severe rate of perinatal morbidity and mortality than newborns of women not diagnosed with COVID-19 (4). Also, a review of 40 different studies from 17 countries found an overall increase in the chances of stillbirth and maternal death during a pandemic (5).

Stillbirths and maternal mortality rates increased by about one-third during the pandemic compared to those before the COVID-19 pandemic. In all studies during the pandemic, the number of women requiring surgery for an ectopic pregnancy increased almost six-fold for ectopic pregnancies during the pandemic versus before the pandemic, and depression also increased (5). It is estimated that approximately 80% of women in childbirth experience mood swings known as day three depression or baby blues. Postpartum depression is considered to be the mildest form of postpartum mood disorder. This depression occurs in the first days after delivery, and the greatest intensity of symptoms occurs between the 3rd and 5th days of the postpartum period (6). After childbirth, women often experience depressed moods, emotional lability, fatigue, tearfulness, tension, and irritability. Postpartum depression does not require treatment. However, since baby blues are a risk factor for the development of postpartum depression, the legitimacy of treating this syndrome as a physiological phenomenon raises doubts (7–10). Postpartum psychosis occurs with a frequency of 0.1% to 0.2% (11).

This type of psychosis is characterized by disorganized thinking, delusional, persecutory or bizarre delusions, visual, tactile, affective lability, cognitive impairment, egosyntonic thoughts, and infanticide (12).

The level of anxiety and fear among pregnant women increased during the COVID-19 pandemic (13).

According to Erikson, hope is “an expectant desire,” a driving force that arises from resolving the first developmental conflict between trust and distrust in the first year of life (14).

For example, people with a high level of basic hope, more often than people with a low level of hope, perceive difficult life situations as a challenge and an opportunity for development. Hope is often activated during illness, which is a medical problem and a psycho-social one. It is a new situation that an individual has to deal with in a certain way. Then a person undertakes an activity defined as coping, aimed at the self-control of emotions and controlling the source of stress (15).

Self-efficacy, a concept originally proposed by the psychologist Albert Bandura, refers to an individual's belief in their capacity to execute behaviors necessary to produce specific performance attainments (16). A person with high self-efficacy views challenges as things that are supposed to be mastered rather than threats to avoid. These people are able to recover from failure faster and are more likely to attribute failure to a lack of effort. Person with a low self-efficacy can have higher levels of stress and depression.

Therefore, we conducted this study to investigate these possible risk factors associated with loneliness, depressive symptoms, anxiety symptoms, among Polish after childbirth women during the COVID-19 pandemic.

## Aim of Study

The study aimed to assess the impact of a pandemic on the mental state of patients after childbirth. The research hypothesis assumed that postpartum women showed a predisposition to postpartum depression due to the pandemic situation, have a reduced level of essential hope and self-efficacy, and an increased level of loneliness.

## Materials and Methods

Three hundred sixty-three postpartum women were included in the study. The research was conducted after obtaining the approval of the Bioethics Committee APK.002.304.2020, from March to September 2021. The study was performed in gynecology and obstetrics two departments in Białystok and one in Biała Podlaska, Poland.

A total of 400 questionnaires were distributed, and 363 fully completed questionnaires qualified for the study. Although the sample selection was deliberate, the study included women who were hospitalized due to past delivery and planned pregnancy on the second day after vaginal delivery or cesarean section in term. Cesarean sections were performed due to lack of progression of labor. The health problems were not analyzed. All subjects tested negative for COVID-19.

The study used the original proprietary questionnaire (seven metric questions and 18 concerning the problem under study) and five standardized scales. The family affluence scale (FAS) reflects the level of material well-being in families. Internal reliability for FAS Cronbach's alpha is 0.643. It consists of several questions and answers evaluated from 0 to 2 points: (1) Does your family own a car or a van? Response categories: no (0 points), yes one or two or more points; (2) Do you have your own bedroom for yourself? no (0 points), yes (1 point); (3) During the past 12 months, did you travel outside your city of residence with your family? Response categories: no, I did not (0 points), once (1 point), twice or more than twice (2 points), and (4) How many personal computers do your family own? none (0 points), one (1 point), two or more than two (2 points); 0–3 points are considered a low FAS score, 4–5 points for the average level, and 6–7 points indicate a high FAS score (17).

The questionnaire described below is the Edinburgh Postnatal Depression Scale (EDPS). The EDPS was developed to identify women who may have postpartum depression. Each answer is given a score between 0 and 3. The maximum score is 30. The 13 or higher points on the EPDS scale are most often used to identify women who might have depression (18, 19). The internal consistency using the Cronbach's alpha coefficient is 0.84.

The Standardized Basic Hope Questionnaire (BHI-12) consists of 12 statements, nine of which are diagnostic, and the remaining three (1, 4, 7) are buffer statements. Diagnostic statements refer to beliefs about the benevolence of the world and the order and predictability of the world. The test person's task is to assess how he agrees with particular statements on a five-point scale. The internal consistency using the Cronbach's alpha coefficient is 0.60–0.81 (20).

The General Self-Efficacy Scale (GSES) is correlated with emotions, optimism, and work satisfaction. Negative coefficients were found for depression, stress, health complaints, burnout, and anxiety. The total score is calculated by finding the sum of all items. For the GSE, the total score ranges between 10 and 40, with a higher score indicating more self-efficacy. Internal reliability for GSE Cronbach's alpha is between 0.76 and 0.90. The higher scores indicate a greater sense of self-efficacy. After conversion to standardized units, the general index was interpreted according to the properties characterizing the sten scale. Results ranging from 1 to 4 stenas were assumed to below, 7 to 10 stenas as high, and 5 and 6 stenas as average (21).

De Jong Gierveld Loneliness Scale (DJGLS) comprises 11 statements, including six items that contain negative sentences describing lack of satisfaction with social contacts, and the remaining five are positive sentences that measure the satisfaction with interpersonal relationships. The respondent was asked to indicate the degree to which the statements express their present situation and feelings. The responses were given on a 5-point scale, from “definitely yes” to “definitely no.” The loneliness index was calculated after re-coding of the 6 “negative” items, and summing up all the test items. The maximum possible score was 55, and higher total scores obtained by the respondent demonstrated a higher sense of loneliness. The Cronbach's alpha—a measure of internal consistency—of the scale is 0.89 (22).

### Statistical Analysis

All statistical analysis was performed with Statistica 13 PL. Results are presented as mean values ± SD. The statistical analysis used the Wilcoxon rank test and chi-square with Yates's correction tests. The critical level for all tests of significance was *p* < 0.05.

## Results

A total of 363 women participated in the study. Analysis using the EPDS suggested suspicion of postpartum depression in 109 women (mean score 15.28 ± 2.22, range 13–20 points), and 254 respondents reported no depression (mean score 6.03 ± 2.63, range 3–11 points). In the following stages, data analysis was divided into women with suspected postpartum depression (group I) and women without suspicion of postpartum depression (group II).

In the first group, the mean age was 33.09 ± 4.70 (24–45 years); in the second group was 32.08 ± 4.6 (23–45). Most of them who were married (84%) had depression, and 82%had no depression. Moreover, the rest were informal (9.17%) or single (6.43%) relationships.

The respondents lived mainly in cities with more than 200,000 inhabitants (35.8%; 36% had depression, and 28% had no depression) and villages (21.1%). About half of respondents had a university education (57% had no depression, and 53% had depression), 25.7% had secondary education (11% had no depression and 33% had depression), 16.5% vocational education, and 4.6% had a bachelor's education.

On the FAS scale, the financial situation was rated at 6.32 ± 2.16 (high level), and in self-assessment, 48.6% were good, 45.9% were very good, and 5.5% were satisfactory. In the second group, the mean age was 32.08 ± 4.58 (22–40 years). Most were also married (81.9%), and the rest were in informal relationships (12.2%) or unmarried (5.9%). The respondents lived mainly in the countryside (33.1%) or in cities with more than 200,000 inhabitants.

About half (57.5%) of the respondents had a university education, 14.2% had secondary education, 5.1% vocational education, and 23.2% had a bachelor's education. On the FAS scale, the financial situation score was 7.11 ± 1.72 (high level). Therefore, 71.7% reported as good, as very good−20.5%, and others had a problem with the declaration.

In group I, in terms of the studied aspects of hospital stay during the pandemic (score on a 0–5 scale), negative features, such as the severity of the need to limit family visits during hospital stay (4.73 ± 0.55 points) and the very fact of hospitalization (4.46 ± 0.76 points) dominated, and in group II, positive features, such as the feeling of emotional support from the family despite the lack of direct contact with family members (3.91 ± 1.57) and emotional support from midwives/nurses (3.25 ± 1.48). Overall, women with depression significantly more often reported selected aspects of hospital stay compared to women without depression. Details are presented in Table 1.

**Table 1:** Selected aspects of a hospital stay during a pandemic.

**During hospitalization**	**A 5-point scale**						***P*** value^*^
	**Group I** **No** **=** **109**			**Group II** **No** **=** **254**			
	**Min**.	**Max**.	**Mean**	**Min**.	**Max**.	**Mean**	
The severity of the need to stay in the hospital	0	5	4.46 ± 0.76	0	5	2.30 ± 1.77	<0.001
The severity of the need to limit family visits	0	5	4.73 ± 0.55	0	5	3.03 ± 2 .02	<0.001
Fear of catching COVID-19 and endangering your own life	0	5	3.42 ± 1.52	0	5	1.43 ± 1.31	<0.001
Fear of becoming infected with COVID-19 and endangering the child's life	0	5	3.85 ± 1.43	0	5	1.81 ± 1.58	<0.001
Taking care of reading. Listening to music or other activities. So as not to think about a pandemic	0	5	2.10 ± 1.77	0	5	1.36 ± 1.72	<0.001
Concentrating on doing something about the situation	0	5	1.63 ± 1.63	0	5	2.22 ± 2.16	0.01
Obtaining emotional support from other patients	0	5	2.90 ± 1.85	0	5	2.93 ± 1.77	NS
Getting emotional support from midwives/nurses?	0	5	2.82 ± 1.78	0	5	3.25 ± 1.48	0.03
Obtaining emotional support from doctors	0	5	2.44 ± 1.90	0	5	2.78 ± 1.40	0.03
Getting emotional support from the family	0	5	4.40 ± 1.21	0	5	3.91 ± 1.57	0.004
Talking/thinking about things which would allow you to escape from the unpleasant feeling	0	5	3.50 ± 1.30	0	5	3.02 ± 1.88	0.01
Attempts to develop an outlining strategy/plan. What needs to be done to survive this period	0	5	2.12 ± 1.78	0	5	0.96 ± 1.40	<0.001
Looking for the good sides in what happened	0	5	3.14 ± 1.62	0	5	2.15 ± 2.03	<0.001
Thinking about escaping from the hospital	0	5	3.32 ± 2.17	0	5	0.32 ± 0.98	<0.001
Thinking. that she could not stand this situation	0	5	3.14 ± 1.98	0	5	0.58 ± 1.04	<0.001
Assessment of hospital care during a pandemic as better_than ever	0	5	2.57 ± 1.88	0	5	2.54 ± 1.75	NS
Assessment of hospital care during a pandemic as worse than ever	0	5	1.92 ± 1.54	0	5	0.64 ± 0.92	<0.001

* *Wilcoxon rank test*.

The respondents from groups I and II did not have family visits very often (94.5% vs. 77.2%) and were most worried about the child's health (95.4% vs. 79.9%). Generally, women with depression and no depression reported in similar percentage negative feelings during the hospitalization Details are presented in Table 2.

**Table 2:** Negative feelings of the respondents concerning the hospitalization* multiple answers possible.

	**Group I** **No = 109**	**Group II** **No = 254**	***P*** value^*^
What do you miss most during your stay in the hospital during the pandemic?			
Family visits	103 94.5%	196 77.2%	NS
Move freely around the hospital premise	26 23.9%	84 33.1%	NS
Reliable information on coronavirus infection	6 5.5%	11 4.3%	NS
Certainty that everything will be fine	26 23.9%	0%	<0.001
It is difficult to say	0%	27 10.6%	0.017
Others	1 person, 0.9%, human empathy	1 person, 0.4%; nothing 1 person, 0.4%, the presence of a husband during childbirth	NS
What are you afraid of the most during your current hospitalization?			
For the health of the child	104 95.4%	203 79.9%	NS
About your health	20 18.3%	33 13%	NS
Here's how a family does without it	25 22.9%	53 20.9%	NS
Others		One person, 0.4%, admission to hospital depending on the test result	

* *Chi^−^square test with Yates's correction*.

Mean values of the scale for measuring the sense of loneliness in group I (27.11 ± 6.00) were significantly (*p* < 0.001) higher than in group II (21.35 ± 7.02). As assessed using the basic hope questionnaire, BHI-12, significantly (*p* < 0.001) lower mean values were obtained in group I (27.92 ± 5.14) than in group II (31.75 ± 4.97). Using the Generalized Self-Efficacy Scale by Schwarzer et al. (23) in group I significantly (*p* < 0.001) lower mean values (28.07 ± 4.86 points) were obtained compared to group II (30.97 ± 3.77 points). The results are presented in Table 3.

**Table 3:** The results of psychometric scales in the group of respondents.

**With suspicion depression** **No** **=** **109**				**Without suspicion depression** **No** **=** **254**				***P*** value^*^
**DJGLS**								
Min.	Max.	Mean		Min.	Max.	Mean		<0.001
19	35	27.11 ± 6.00		10	35	21.35 ± 7.02
**BHI-12**								
Min.	Max.	Mean		Min.	Max.	Mean		<0.001
15	35	27.92 ± 5.14		21	40	31.75 ± 4.97		
**GSES**								
Min.	Max.	Mean point	Mean sten	Min.	Max.	Mean point	mean sten	<0.001
18 points	38 points	28.07 ± 4.86		20 points	40 points	30.97 ± 3.77		
2 sten	20 stens		5.87 ± 1.96	3 sten	10 sten		6.02 ± 1.38	

* *Wilcoxon rank test*.

The results of the analysis of the relationship between selected aspects of hospital stay during a pandemic and psychometric scales are presented in Table 4.

**Table 4:** Relationships between selected aspects of a hospital stay during a pandemic and psychometric scales.

**Problem**	**Group**	**Scale/*****p*** **value**			
		**FAS**	**DJGLS**	**BHI-12**	**GSES**
The severity of the need for hospitalization during the pandemic	I	NS	*R* = 0.266 *p* = 0.005	NS	NS
	II	NS	*R* = −0.179 *P* = 0.004	*R* = 0.132 *P* = 0.03	NS
The severity of limiting family visits	I	NS	NS	NS	NS
	II	NS	NS	*R* = −0.197 *P* = 0.039	NS
Fear of catching COVID-19 and endangering your own life	I	NS	NS	NS	NS
	II	*R* = 0.201 *P* = 0.0012	NS	*R* = −0.236 *P* ≤ 0.001	NS
Fear of becoming infected with COVID-19 and endangering the child's life	I	NS	NS	NS	NS
	II	NS	NS	*R* = −0.206 *P* <0.001	*R* = −0.126 *P* = 0.04
Emotional support from other patients	I	NS	NS	NS	NS
	II	NS	*R* = 0.139 *P* = 0.026	NS	*R* = 0.205 *P* <0.001
Emotional support from midwives/nurses	I	NS	NS	NS	NS
	II	*R* = 0.192 *P* = 0.002	NS	NS	*R* = −0.199 *P* = 0.001
Emotional support from doctors	I	NS	NS	*R* = −0.202 *P* = 0.03	NS
	II	*R* = 0.300 *P* <0.001	NS	*R* = 0.124 *P* = 0.04	NS
Emotional support from the family	I	NS	NS	NS	NS
	II	*R* = 0.202 *P* <0.01	NS	NS	NS
Thoughts to escape the hospital	I	NS	NS	NS	NS
	II	*R* = −0.202 *P* = 0.01	NS	NS	NS
He thinks he cannot handle this situation	I	NS	NS	NS	NS
	II	*R* = −0.303 *P* <0.001	*R* = 0.271 *P* <0.001	NS	NS
Feeling of better hospital care than ever during a pandemic	I	NS	*R* = 0.139 *P* = 0.02	NS	NS
	II	NS	NS	*R* = 0.127 *P* = 0.04	R = 0.251 <0.001
Feeling of inferior hospital care during a pandemic than ever	I	NS	NS	NS	NS
	II	NS	*R* = 0.367 *P* <0.001	*P* = −0.136 *P* = 0.03	*P* = 0.259 *P* <0.001

In both groups, statistically significant relationships were found between several parameters:

- the severity of the need for hospitalization during the pandemic and the severity of the feeling of loneliness, and in group II, from the level of basic hope,
- emotional support from doctors and basic hope and in the second group, also from the financial situation,
- a sense of better hospital care during a pandemic than usual, a sense of loneliness in group I, and the level of basic hope and self-efficacy in group II,

- the severity of limiting family visits and the level of basic hope,
- fear of becoming infected with COVID-19 and endangering one's own life in addition to their financial situation and the level of basic hope,
- fear of contracting COVID-19 and threatening the child's life and the level of basic hope and self-efficacy,
- emotional support from other patients as a measure of loneliness and self-efficacy,
- emotional support from midwives/nurses versus financial situation and self-efficacy,
- emotional support from the family and financial situation,
- thinking about escaping from hospital and the financial situation,
- thinking that you will not be able to cope with the current situation and the financial situation and feelings of loneliness,
- feeling that hospital care is worse than ever during a pandemic, and a sense of loneliness, the level of essential hope and self-efficacy.

## Discussion

The aim of this study was to assess the impact of pandemic COVID-19 on the mental state of women after childbirth. In the present study as much as 30% of the respondents showed a risk of postpartum depression. The most felt was the limitation of family visits during the hospital stay. The feeling of loneliness in women with depression was higher, and hope and generalized self-efficacy were lower than in women without depression. The differences between these relationships were statistically significant.

Childbirth is generally a very stressful life situation (24). Almost all women experience anxiety, including mild anxiety in about 80% of pregnant women, and about 20% of women experience intense anxiety. Anxiety intensifies in the third trimester of pregnancy, during which 6%−10% of women struggle with very severe pathological anxiety, while 2% of women are diagnosed with extreme anxiety due to childbirth (25, 26).

In an Irish study, after a month of forced isolation 44% of pregnant women had a depressed mood, 14% because of a deterioration in the financial situation due to lack of work, 11% because of tensions with family members, and 4% because of deterioration in the relationship with a partner. Another Irish study demonstrated that before the COVID-19 pandemic, 83% of pregnant women were not worried about their health. Since the pandemic, 50.7% of them were worried about it all the time, 35% isolated themselves for fear of the virus, and 32% worked at home (27).

The above studies confirm our findings. As the most severe factors during hospitalization during the pandemic, the respondents indicated the need to limit family visits during the hospital stay, the mere fact of staying in the hospital, the child becomes infected with COVID-19, and the fear of their own health status.

Sade et al. (28) assessed the risk of depression among pregnant women hospitalized during the COVID-19 pandemic compared with women hospitalized before the COVID-19 pandemic. All participating women completed the EPDS. Women hospitalized during the period of strict COVID-19 isolation had a relative risk of obtaining a high (> 10) EPDS score compared to women hospitalized before the COVID-19 pandemic (*p* = 0.498).

In the present study, the EPDS scale was also used. It was revealed that in 109 subjects in 30.2% of the risk of postpartum depression may be suspected. Durankuş and Aksu obtained similar results (29). They stated that among pregnant women, 35.4% had a result above 13 in the EPDS. The authors also found a statistically significant effect of COVID-19 on mental health, social isolation, and mean scores in the Beck Depression Inventory and Beck Anxiety Inventory (BDI and BAI, respectively). In a study from Italy among 281 mothers, symptoms of depression were found in 26% and anxiety in 32% of the respondents. Mothers who reported no exposure to SARS-CoV-2 during pregnancy and those who reported at least one direct or indirect exposure did not differ in affective symptoms (30).

Also, Khamees et al. evaluated the level of anxiety and depression (using EPDS) in a group of 120 pregnant women. Nulliparous and multiparous women had a relatively high probability of developing depression (31).

Future mothers and those who have just given birth experience more significant stress, experience greater anxiety, and more severe symptoms of depression. The cesarean section increases the occurrence of postpartum depression and makes it difficult for mothers to bond with their babies, leading to depression in the whole family. These results were confirmed in the present study. In the group indicating a risk of depression, the mean values of loneliness were higher than those without risk.

The current study examined how women evaluate the support they receive after childbirth. It was revealed that the emotional support received from midwives/nurses and doctors caused a noticeable decrease in the risk of postpartum depression in the respondents, and the respondents at risk of postpartum depression were viewed better by the family.

Yali and Lobel conducted a longitudinal study in a group of 163 pregnant women between 10 and 25 and 21 and 35 weeks of pregnancy. It was assumed that stress resistance focuses on resources that facilitate adaptation under stressful circumstances. Positive self-esteem was the only coping strategy that produced less stress. However, analyses show that these “resources” are only related to stress in early pregnancy and that coping is not related to stress throughout pregnancy. The results suggest a high level of stability in coping with stress throughout pregnancy (32).

In this context, hope, treated as emotion and associated with commitment and coping with life difficulties or waiting to fulfill one's desires, also gains significance (33).

Research by Trelak and Demkiewicz (34) demonstrated that hope might be one of the factors explaining the attitudes of future mothers toward motherhood. In their opinion, a strong sense of hope influences the behavior and emotions of women, contributing to adequate identification with a new role, the role of the mother.

In the present study, a lower level of hope had women with suspicion of depression. It was revealed that women with weak and robust hope statuses differed in coping strategies. On the other hand, the lowest results on the displacement scale (ignoring the problem) were preferred by women during the last weeks of pregnancy. A high level of hope among women at the beginning of pregnancy resulted in a more frequent choice of strategies.

It is believed that a higher sense of self-efficacy leads to an increase in motivation to act and is associated with better achievements of an individual. Therefore, the self-concept in health psychology is the essential determinant of establishing and introducing changes in health behaviors. The stronger beliefs about self-efficacy, the higher the goals people set for themselves, and the stronger their commitment to the intended behavior even in mounting failure was important. Low self-efficacy is associated with depression, anxiety, and helplessness (23, 33, 34).

Rogala's and Ossowski's research included 234 women during weeks 38 to 42 of pregnancy with a normal course. It was shown that they have a high level of generalized efficacy (30.19), which was higher than the Polish average (27.32). No correlation was found between the level of self-efficacy and the duration of labor, labor activity, the number of painkillers used, and the method of delivery completion. However, women who gave birth to children in good condition assessed their competencies highly, were satisfied with the care of the staff, and had a higher sense of self-efficacy (35).

In the present study, the values of generalized self-efficacy in the group at risk of labor depression were lower than in the group without the risk of postpartum depression.

In the 1980s, Hopkins et al. performed a critical analysis of 110 research papers and distinguished three forms of clinical depression during this period: (1) postpartum depression (or baby blues), (2) postpartum depressive psychoses, and (3) depressive syndromes of varying severity. In addition, these disorders differed in the type and severity of symptoms and the time of onset and duration (36).

In the current research, correlations between the severity of the need for hospitalization during the pandemic and the feeling of loneliness. Emotional support from doctors, essential hope, and a sense of better hospital care during the pandemic were more important than usual.

However, it is known that a good pregnancy and an adequately conducted childbirth are not in themselves the cause of depressive disorders but may be associated with the risk of various factors that cause mental disorders.

Therefore, early identification of potential risks for postpartum mood disorders should include sociodemographic evaluation, personality, psychiatric history of the woman, recent life events, and past and present obstetric-gynecological factors (37).

### Study Limitations

First, the sample size is not too big, which can affect the result of a type II error in statistical analysis. Second, the present findings should be generalized cautiously since they were drawn from a non-randomized sample and obtained through screening measures. Third, the cross-sectional nature of the study significantly limits causal explanations.

## Conclusions

In the studied group of women after childbirth, as much as 30% of respondents indicated the risk of postpartum depression.

The group at risk of postpartum depression (I) reported that family visits were limited during the hospital stay.

In the group without the risk of depression (II), respondents reported emotional support from the family despite the lack of direct contact with her and emotional support from midwives/nurses.

The feeling of loneliness in group I was higher, and hope and generalized self-efficacy were lower than in group II, and the differences between these relationships were statistically significant.

Postpartum depression is significantly underdiagnosed and undertreated. Therefore, early depression prevention and monitoring of women after childbirth is an important issue. Further studies on the larger groups are needed to confirm these findings. We suggest testing for early detection of depression in women four weeks after delivery.

## Data Availability Statement

The original contributions presented in the study are included in the article/supplementary material, further inquiries can be directed to the corresponding author.

## Ethics Statement

The studies involving human participants were reviewed and approved by the Bioethics Committee APK.002.304.2020 Medical University of Bialystok. The patients/participants provided their written informed consent to participate in this study.

## Author Contributions

AK-B created the research concept. AK-B, MM-G, GB, and AS were collecting materials to the study, prepared the initial version of the manuscript, which was corrected, supervised, and completed by NW. AK-B conducted data analysis. All authors contributed to the article and approved the submitted version.

## Conflict of Interest

The authors declare that the research was conducted in the absence of any commercial or financial relationships that could be construed as a potential conflict of interest.

## Publisher's Note

All claims expressed in this article are solely those of the authors and do not necessarily represent those of their affiliated organizations, or those of the publisher, the editors and the reviewers. Any product that may be evaluated in this article, or claim that may be made by its manufacturer, is not guaranteed or endorsed by the publisher.
